# Cyclic Superelasticity, Elastocaloric Effect, and Shape Memory Effect of Solution-Treated Ti_50_Ni_41_Cu_7_Co_2_ Alloy

**DOI:** 10.3390/ma18245489

**Published:** 2025-12-05

**Authors:** Niranjan Kumar Choudhry, Da-Syuan Chou, Chih-Hsuan Chen

**Affiliations:** 1 Department of Mechanical Engineering, National Taiwan University, Taipei 106, Taiwan; niranjan2025@ntu.edu.tw (N.K.C.);; 2 Department of Materials Science and Engineering, National Taiwan University, Taipei 106, Taiwan

**Keywords:** shape memory alloy, superelasticity, shape memory effect, elastocaloric effect

## Abstract

**Highlights:**

**What are the main findings?**

- Superelastic cycle test transformed the stress–strain curve into quasi-linear one with homogeneous strain.
- SME of 6.21% recoverable strain with 0.44% irrecoverable strain was obtained at a bias stress of 300 MPa.
- After training, transformation strain and elastocaloric effect became stable at 1.3%, and −4.3 K.
- A recoverable strain of about 0.5% was still achievable from up to 443 K.

**What are the implications of the main findings?**

- Solution-treated Ti_50_Ni_41_Cu_7_Co_2_ SMA exhibited stable functionalities after 100 cycles.
- Cyclic superelasticity test significantly stabilizes the residual martensite even up to 573 K.
- Further improvement of the performance can be achieved by alloy design and microstructural control.

**Abstract:**

In recent years, there has been an increasing interest in studying multi-component alloys. A bulk solution-treated Ti_50_Ni_41_Cu_7_Co_2_ SMA was prepared and investigated. The functional properties, including phase transformation temperature, shape memory effect, cyclic superelasticity, and elastocaloric response, were systematically evaluated. The alloy exhibited a M_s_ temperature of around 250 K, which is beneficial for applications at room temperature. Shape memory effect with a maximum recoverable strain of 6.21% was obtained under a biased stress of 300 MPa. The superelasticity rapidly became stable during the cyclic test, reducing irrecoverable strain from 2.8% to 0.01% by the 10th cycle. After 250th superelastic cycles, the alloy exhibited a stable recoverable strain of 1.3%, and a lower critical stress for transformation (270 MPa, down from 405 MPa). The elastocaloric cooling effect reached −4.9 K at the 50th cycle and stabilized at −4.3 K thereafter. With an increase in operating temperature, the elastocaloric effect diminished and disappeared above 383 K, and the SMA retained a notable recoverable strain of ~0.5% up to 443 K.

## 1. Introduction

Shape memory alloys (SMAs) are metallic functional materials exhibiting potential in advanced engineering applications, including actuators, biomedical implants, cooling devices, and so on, due to their exceptional superelasticity, elastocaloric cooling effect, and shape memory effect [1,2,3]. Among SMAs, TiNi-based SMAs are extensively studied, and different alloying systems like binary, ternary, and quaternary are proposed [4,5]. In recent years, the TiNiCuCo quaternary alloying system has gained more attention due to the combination of the beneficial effects of Cu and Co. Cu helps in the stabilization of martensite, improves thermal cyclic stability, and reduces hysteresis [6,7]; while Co tunes transformation temperature and improves functional fatigue resistance [8,9,10]. Therefore, a quaternary alloying of TiNiCuCo serves the advanced functional requirements for a broader application range. Curtis et al. [11] developed freestanding Ti_53.3_Ni_30.9_Cu_12.9_Co_2.9_ thin-film superelastic alloys for stretchable interconnects used in flexible electronic systems. They proposed Cu/TiNiCuCo/Cu serpentine composites, which showed superior mechanical and electrical conductivity. Ahmadi et al. [12] studied the influence of vibration (both free and forced) on TiNiCuCo for understanding its potential application in miniature scale damping. They developed a thermodynamics-based finite element model and simulated the fraction of martensite phase during load-induced transformation. They examined the effect of different variables, such as pre-strain, strain rate, and excitation load, on the damping energy. They obtained a damping capacity of 0.17. Ossmer et al. [4] studied the effect of tensile load cycles on the elastocaloric cooling effect of the Ti_54.7_Ni_30.7_Cu_12.3_Co_2.3_ and Ti_55_Ni_29.6_Cu_12.6_Co_2.8_ films. They highlight a correlation between strain and temperature bands for the TiNiCuCo film. Xu et al. [5] used a thin film of TiNiCuCo to develop a cascaded elastocaloric device for providing a larger cooling capacity up to 900 mW by employing a series of Ti_55_Ni_29.6_Cu_12.6_Co_2.8_ films. Ti-rich Ti_54.7_Ni_30.7_Cu_12.3_Co_2.3_ shape memory thin films [13] were reported to exhibit ultrahigh-stable functionality due to the existence of nano-scale Ti_2_Cu phase, which accommodates the interfacial strain between the B2 and B19 during phase transformation. The presence of nanoscale Ti_2_Cu precipitates in bulk TiNiCuCo SMAs improves their tensile cycle stability [14].

Even though TiNiCuCo alloys possess promising characteristics [5,15], limited research has been conducted for the bulk TiNiCuCo SMAs. Existing studies have primarily focused on transformation behavior and mechanical responses under restricted conditions, however, leaving significant gaps in understanding the generation and propagation of local strain bands, temperature profiles, and phase transformation of bulk TiNiCuCo SMAs during and after the cyclic superelasticity test. Furthermore, there is a need to study the local presence of these features and their impacts on superelasticity, elastocaloric effect, and shape memory effect (SME).

In this study, a bulk material of Ti_50_Ni_41_Cu_7_Co_2_ was prepared, in which the Ti content was designed to be 50 at. % to avoid the excessive formation of the brittle Ti_2_Ni phase. A systematic investigation was conducted for phase transformation, superelasticity, and elastocaloric effect. Particular emphasis was placed on understanding the local distributions of phase transformation and elastocaloric cooling during and after the superelastic cycles. These results aim to provide fundamental insights into the design of multi-component SMAs and to highlight their suitability for emerging technologies requiring both reliability and efficiency.

## 2. Materials and Methods

An ingot of approximately 138 g of Ti_50_Ni_41_Cu_7_Co_2_ was prepared from 99.99 wt. % pure raw materials of Ti, Ni, Cu, and 99.98 wt. % of Co using an arc remelting furnace. The Ti oxygen-absorbing ingot was smelted twice to absorb the remaining oxygen present in the melting furnace before melting the alloy. The homogeneous distribution of metal components was ensured by melting the prepared alloy six times and smelting twice into the molding mold. An ingot with dimensions of approximately 70 × 40 × 10 mm^3^ was obtained. The ingot thickness was reduced to 2 mm through hot rolling at 1173 K. Solution treatment of the hot-rolled plate was performed at 1173 K for an hour in an air furnace, followed by quenching in ice water. Scanning electron microscopy (SEM) was employed to investigate the microstructural features. A differential scanning calorimeter (DSC 25, TA Instruments, New Castle, DE, USA) was used to measure the phase transformation temperatures at a cooling/heating rate of 10 K/min. The storage modulus and loss factor (tan(δ)) were measured at a frequency of 1 Hz and an amplitude of 10 μm using a dynamic mechanical analyzer (DMA, TA 2980, TA Instruments, New Castle, DE, USA). DMA was also employed to evaluate the shape memory effect with a cooling/heating rate of 5 K/min. The shape memory effect test was conducted under a three-point bending mode on a specimen measuring 30 × 1.5 × 0.7 mm^3^ in size. A bias stress of 50 MPa, 100 MPa, 150 MPa, 200 MPa, 250 MPa, and 300 MPa was applied, and the corresponding strain-temperature curve was obtained for each cycle.

The microstructural observations were performed using a field-emission scanning electron microscope (FESEM, JSM-7800F, JEOL, Tokyo, Japan) equipped with an energy-dispersive spectroscopy (EDS) and electron backscatter diffraction (EBSD) detectors. The diffraction spectra were collected with an X-ray diffractometer (XRD, Discovery D8, Bruker, Mannheim, Germany) equipped with a cooling stage.

Tensile superelasticity and elastocaloric cooling effect were measured using a Shimadzu AG-IS 50 kN universal testing machine (Tokyo, Japan) equipped with an environmental furnace (TEC-N300). The gauge size for the tensile test was 8 mm × 8 mm × 2 mm. The tensile specimens were prepared by mechanical polishing first, and then high-temperature-resistant black paint was applied. Finally, high-temperature-resistant silver paint was sprinkled over it. The quasi-static superelasticity tests were performed at a strain rate of 5 × 10^−4^ s^−1^. Digital image correlation (VIC-2D DIC) was employed to record deformation and strain of the test specimens during mechanical testing, while strain distribution was analyzed using Correlated Solutions VIC-2D 6 software afterward. The elastocaloric effect of Ti_50_Ni_41_Cu_7_Co_2_ SMA was recorded using the FLIR A615 infrared thermal imager (FLIR, Shanghai, China) at 200 FPS. During loading, a constant strain rate of 3 × 10^−4^ s^−1^ was applied until a maximum load of 425 MPa was reached. Thereafter, the load was held for 50 s, and then rapidly unloaded at a higher strain rate of 5 × 10^−1^ s^−1^ to achieve a near adiabatic condition during the reverse phase transformation.

## 3. Results and Discussion

### 3.1. Characterization of Solution-Treated Ti_50_Ni_41_Cu_7_Co_2_ SMA

**Figure 1:**
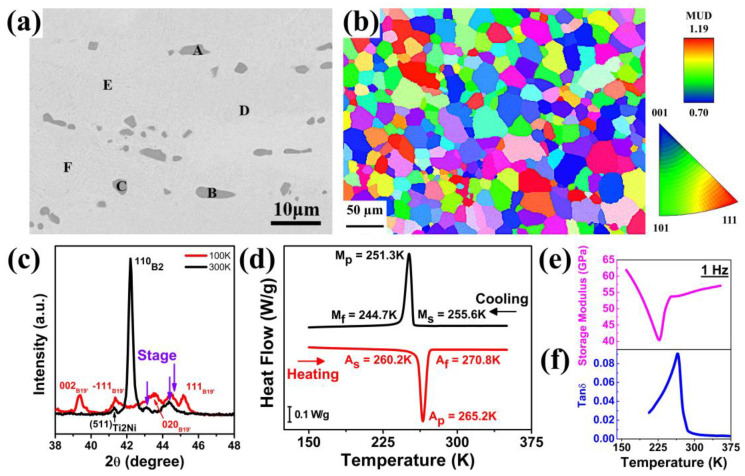
Characterization of solution-treated Ti_50_Ni_41_Cu_7_Co_2_ SMA; (**a**) SEM backscattered electron image, (**b**) crystal orientation distribution diagram, (**c**) XRD diffraction spectrum, (**d**) DSC test results with cooling and heating curve; DMA test results (**e**) storage modulus and (**f**) tan(δ).

Figure 1a shows the SEM backscattered electron image of Ti_50_Ni_41_Cu_7_Co_2_ after solution treatment at 1173 K for an hour. The compositions at different locations were analyzed by energy-dispersive spectroscopy (EDS) and are presented in Table 1. The dark gray precipitates have an average composition of 65.3 at. % Ti, 29.1 at. % Ni, 4.8 at. % Cu, and 0.8 at. % Co while an average composition of base matrix is 49.0 at. % Ti, 40.2 at. % Ni, 8.9 at. % Cu, and 1.9 at. % Co. The lower Ti content in the matrix is mainly due to excess Ti in the Ti_2_(Ni,Cu) precipitates. The decrease in Ni and increase in Cu are possibly attributed to the overlap of Cu and Ni signals in EDS, which reduces compositional accuracy and causes deviations from the expected values. Additionally, the low Cu content in the Ti_2_(Ni,Cu) phase also caused an increase in the Cu content in the matrix. These dark gray Ti_2_(Ni,Cu) particles were observed embedded in the matrix, which were inevitably formed due to the presence of even a small amount of oxygen during the melting, hot-rolling, and solution treatment process [16,17]. The oxygen is a Ti_2_Ni (i.e., the Ti_2_(Ni,Cu) phase in this study) stabilizer [18,19], which induces the formation of Ti_2_(Ni,Cu) phase in this study, even though the alloy is not rich in Ti (i.e., Ti is not above 50 at. %), as also reported in several studies [17,20,21,22].

Figure 1b shows the electron backscattered diffraction (EBSD) image of the ST sample, highlighting its distribution of crystal orientations. The observation direction was perpendicular to the rolling direction. Using the intercept method, the average grain size was estimated to be approximately 30 μm. Additionally, although the crystals are predominantly oriented along the [111] direction, their intensity was not significant (1.19). Therefore, the alloy exhibited a weak texture and was significantly influenced by the orientation.

Figure 1c shows the XRD spectra collected at 300 K and 100 K. At 300 K, the B2 parent phase and Ti_2_Ni were identified on the spectrum. Because the second phase exhibited a cubic structure (crystal structure of Ti_2_Ni) and contained a slight amount of Cu content (Table 1), it was marked as the Ti_2_(Ni,Cu) phase. After cooling to 100 K, the B2 parent phase transformed to B19′ martensite, as identified by the diffraction signals. The XRD spectra confirmed that the alloy underwent a B2 to B19′ martensitic transformation.

A DSC heat flow curve was used to determine the characteristic martensitic temperatures, including martensite start temperature (M_s_), martensite peak temperature (M_p_), martensite finish temperature (M_f_), austenite start temperature (A_s_), austenite peak temperature (A_p_), and austenite finish temperature (A_s_), as shown in Figure 1d. A sharp phase change peak during cooling and heating confirmed an obvious phase transformation B2 ⇿ B19′. The M_p_ and A_p_ were 251.3 K and 265.2 K, respectively. Furthermore, M_s_ and M_f_ were 255.6 K and 244.7 K, and A_s_ and A_f_ were 260.2 K and 270.8 K, respectively, with a transformation temperature range of 10.9 K for martensitic transformation (M_tr_ = M_s_ − M_f_) and 10.6 K for reverse martensitic transformation (A_tr_ = A_f_ − A_s_).

The variations in storage modulus and tan(δ) with temperature were measured by DMA, as shown in Figure 1e,f, respectively. With a rise in temperature, the storage modulus fell sharply, reaching a minimum at a temperature of 265 K, and then rose steeply. Valley formation at a temperature of 265 K corresponded to the phase transformation (martensite ⇿ austenite). During the phase transformation, the storage modulus softens temporarily due to rearrangements in the crystal structure (coexistence of both martensite and austenite phases). However, tan(δ) increased with temperature, reaching a maximum at a temperature of 266 K, and then fell sharply, forming a peak at a temperature of 266 K. The peak formation also confirmed the phase transformation within the material, as this peak formation showed more internal friction generation, mainly due to interface movement during the phase transformation. The thermal analyses by DSC and DMA showed that the crystal structure transformed with temperature, which was a characteristic of SMAs.

### 3.2. Shape Memory Effect via Thermal Cycling Test Under Bias Applied Stress

**Figure 2:**
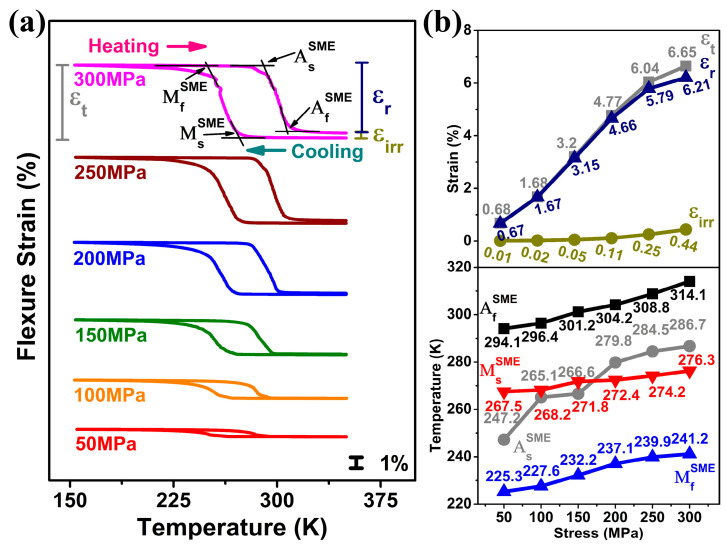
Shape memory effect at different applied stress conditions; (**a**) shape memory strain-temperature curves, (**b**) dependence of various strains: ε_t_ (maximum strain), ε_r_ (recoverable strain), and ε_irr_ (irrecoverable strain), and transformation temperatures on the applied stress.

Shape memory effect (SME) was studied for different applied stress conditions (50 MPa to 300 MPa) in a temperature range of 150 K to 350 K. Figure 2a shows the strain–temperature curves, which include cooling stage (austenite → martensite) and heating stage (martensite → austenite) for a given stress condition. The various strains measured during experiments were maximum strain (ε_t_), recoverable strain (ε_r_), and irrecoverable strain (ε_irr_), as indicated in Figure 2a. The results are summarized in Figure 2b. In Figure 2b, as the applied stress increased from 50 to 300 MPa (in a step of 50 MPa), all three strains increased; ε_t_ increased from 0.68% to 6.65%, ε_r_ from 0.67% to 6.21%, and ε_irr_ from 0.01% to 0.44%. Maximum strain (ε_t_) and recoverable strain (ε_r_) were nearly equal up to 200 MPa, but they deviated as stress increased beyond 200 MPa, indicating the occurrence of plastic deformation during martensitic transformation under this applied stress level. As shown in Figure 2b, higher stresses triggered not only martensite formation and reorientation but also dislocation generation and plastic deformation, resulting in increased irrecoverable strain (ε_irr_) and reduced SME efficiency. Further, increasing the applied stress steepens the cooling and heating slopes, thereby narrowing the transformation temperature range. Additionally, it was noted that the transformation temperatures under applied stress, including $M_{s}^{SME}$, $M_{f}^{SME}$, $A_{s}^{SME}$ and $A_{f}^{SME}$, increased with increasing applied stress, following the Clausius–Clapeyron relation.

### 3.3. Cyclic Tensile Superelasticity

**Figure 3:**
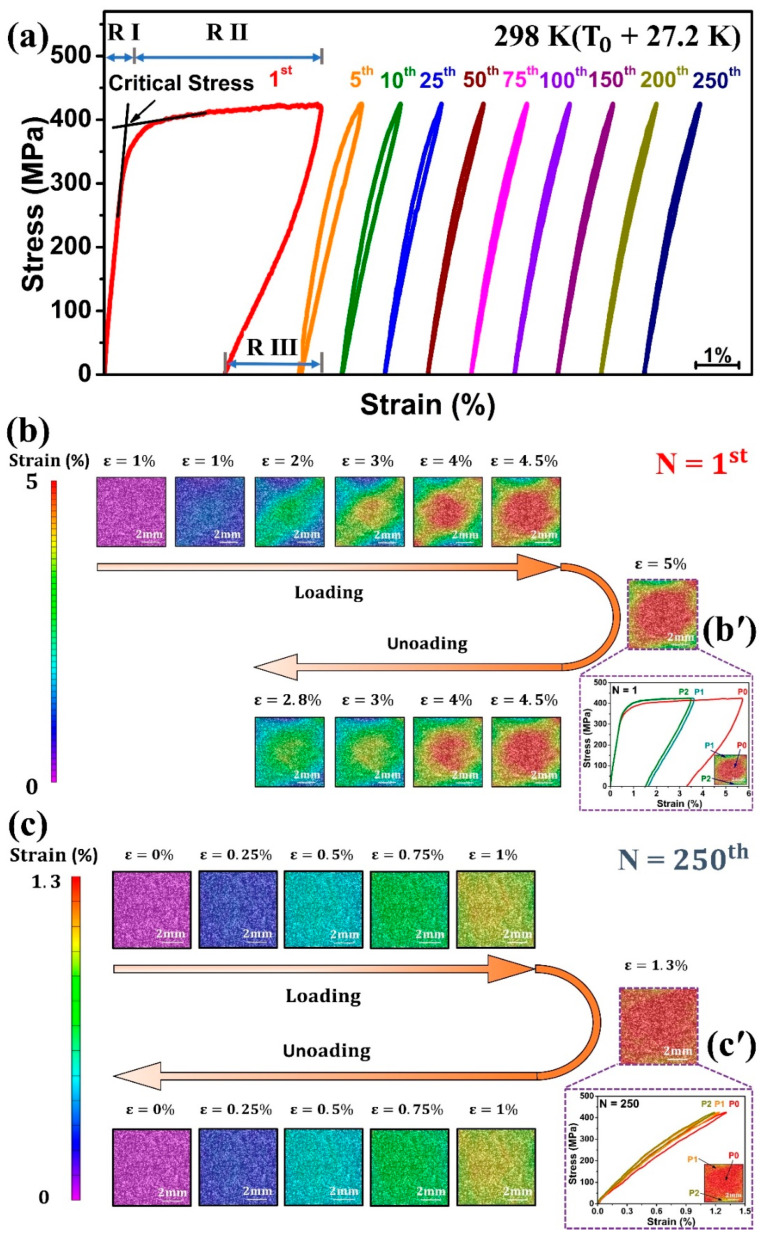
(**a**) Cyclic tensile superelasticity at 425 MPa; DIC analysis of 1st cycle (**b**) strain mapping during loading and unloading, and appearance of Lüders-like strain map (**b’**) local strain distribution at maximum strain and the associated stress–strain curves. DIC analysis of the 250th cycle (**c**) strain mapping during loading and unloading, and (**c’**) local strain distribution at maximum strain and the associated stress–strain curves.

Cyclic tensile superelasticity test was conducted with a constant maximum stress of 425 MPa at room temperature (298 K), which is higher than A_f_ (270.8 K). The stress–strain curve during loading and unloading of the 1st cycle can be distinguished into three main regions, as shown in Figure 3a. Region I (R I): austenite (B2) phase deformed elastically showing linear stress–strain response; region II (R II): this region started when stress reached to the critical stress, leading to the transformation of austenite (B2) into martensite (B19′); region III (R III): the unloading process caused reverse transformation with almost linear fall in stress–strain curve. The maximum strain (ε_t_), recoverable strain (ε_r_), and irrecoverable strain (ε_irr_) obtained during the first cycle were 5.0%, 2.2%, and 2.8%, respectively. An obvious residual strain was observed during the 1st superelastic cycle.

Figure 3b shows the DIC analyses for the 1st cycle. During loading, Lüders-like strain bands appeared, and a change in strain was concentrated at the specimen center. During unloading, significant residual strain remained due to extensive B2 → B19′ transformation. The two lattices, B2 and B19′, generated dislocations at their interfaces, which hinder the reverse transformation [14]. As a result, residual martensite was left [23], causing residual strain after unloading. Figure 3b’ illustrates the local stress–strain distribution at the maximum strain (ε_t_ = 5%) of the 1st cycle. Even though the average strain was 5%, the strain distribution throughout the region of consideration was non-uniform. The center region (P0) underwent the highest strain of 5.7%; however, regions (P1 & P2) far away from the center were less strained, reaching 3.6% and 3.5%, respectively, confirming a localized and heterogeneous transformation process.

The high irrecoverable strain indicated its lower strength and was prone to plastic deformation, which was undesirable for engineering applications. To achieve practical applications, stable superelasticity and elastocaloric performance were obtained through cyclic tensile stretching to 425 MPa at room temperature. Figure 3a shows the cyclic tensile response of solution-treated Ti_50_Ni_41_Cu_7_Co_2_ SMA. The stress–strain curve changed from the flag-type mode (1st cycle) to a quasi-linear one after tensile superelasticity cycles. In the first cycle, the maximum strain was 5.0% and an irrecoverable strain was 2.8%, but with an increase in the number of cycles, these strains decreased, and by the 10th cycle, their values decreased to 1.4% and 0.01%, respectively. For the later cycles, the maximum strain (ε_t_) and recoverable strain (ε_r_) stabilized at 1.3% while irrecoverable strain almost vanished, indicating the reaching of a stable status.

Figure 3c shows the DIC analyses of the 250th superelastic cycle. The strain distributions during the loading and unloading were more uniform than in the 1st cycle, and no residual strain was observed. Local strain distribution at the maximum strain of the 250th cycle is shown in Figure 3c’. It can be seen that when P0 reached the maximum strain of 1.31%, P1 was 1.24% and P2 was 1.19%, indicating that the defects or dislocations generated during previous cycles promote the homogeneous martensitic phase transformation [24].

**Figure 4:**
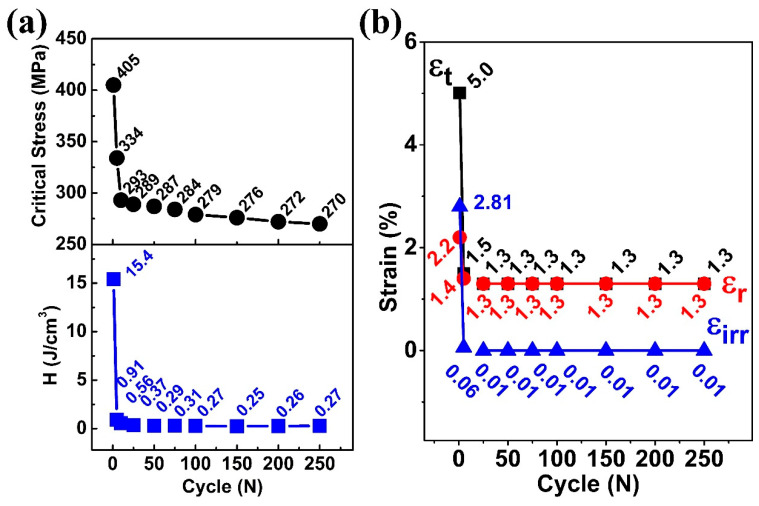
Evolutions of (**a**) critical stress for stress-induced martensitic transformation and dissipated energy (H), and (**b**) the ε_t_ (maximum strain), ε_r_ (recoverable strain), and ε_irr_ (irrecoverable strain) with the superelastic cycle.

Figure 4a summarizes the evolution of the critical stress during the superelastic cycles. Critical stress for a shape memory alloy is defined as the stress that needs to be applied for the start of transformation from austenite (B2) to martensite (B19′) at a given temperature. Generally, the critical stress is identified as an intersection point between the tangents to the linear elastic deformation region I (R I) and the plateau transformation region II (R II), as shown in Figure 3a. The critical stress for martensitic transformation dropped from 405 MPa in the 1st cycle to 334 MPa at the 5th and 270 MPa at the 250th cycles, as shown in Figure 4a. This reduction in critical stress during the cyclic test was attributed to the residual martensite, which generated residual strain fields that promote transformation, thereby lowering the stress required to induce martensitic transformation [25]. Further, the dissipation energy (H), which was calculated from the area of the stress–strain curves, also decreased sharply from 15.4 J/cm^3^ in the 1st cycle to 0.91 J/cm^3^ in the 5th and 0.27 J/cm^3^ in the 250th cycles. This decline in dissipation energy indicated that the friction between the austenite and martensite during martensitic transformation reduced, associated with the quasi-linear stress–strain behavior.

The evolutions of the maximum strain (ε_t_), recoverable strain (ε_r_), and irrecoverable strain (ε_irr_) during the superelastic cycles are summarized in Figure 4b. It can be seen that when the maximum strain at the 1st cycle was 5.0%, the irrecoverable strain was 2.8%, which dropped sharply by 97.8% to 0.06% in the first 5 cycles. A similar observation was found for the maximum strain (ε_t_). For the first 5 cycles, the maximum strain dropped by 70%, from 5.0% to 1.5%. In the 10th cycle, the maximum strain (ε_t_) and irrecoverable strain (ε_irr_) became 1.3% and 0.01%, respectively. After the 25th cycle, irrecoverable strain became negligible (0.01%), and the recoverable strain (ε_r_) reached the maximum strain (ε_t_), indicating that the system became stable. One can notice that even though the same maximum stress was reached during each cycle, the maximum strain attained during the loading decreases from 5% in the first cycle to 1.3% during the 250th cycle, indicating that the residual deformation during the cycle reduced the maximum strain by inhibiting the martensitic transformation. However, at the same time, the stability of the superelastic behavior became quasi-linear and stable.

### 3.4. Cyclic Elastocaloric Cooling Effect

**Figure 5:**
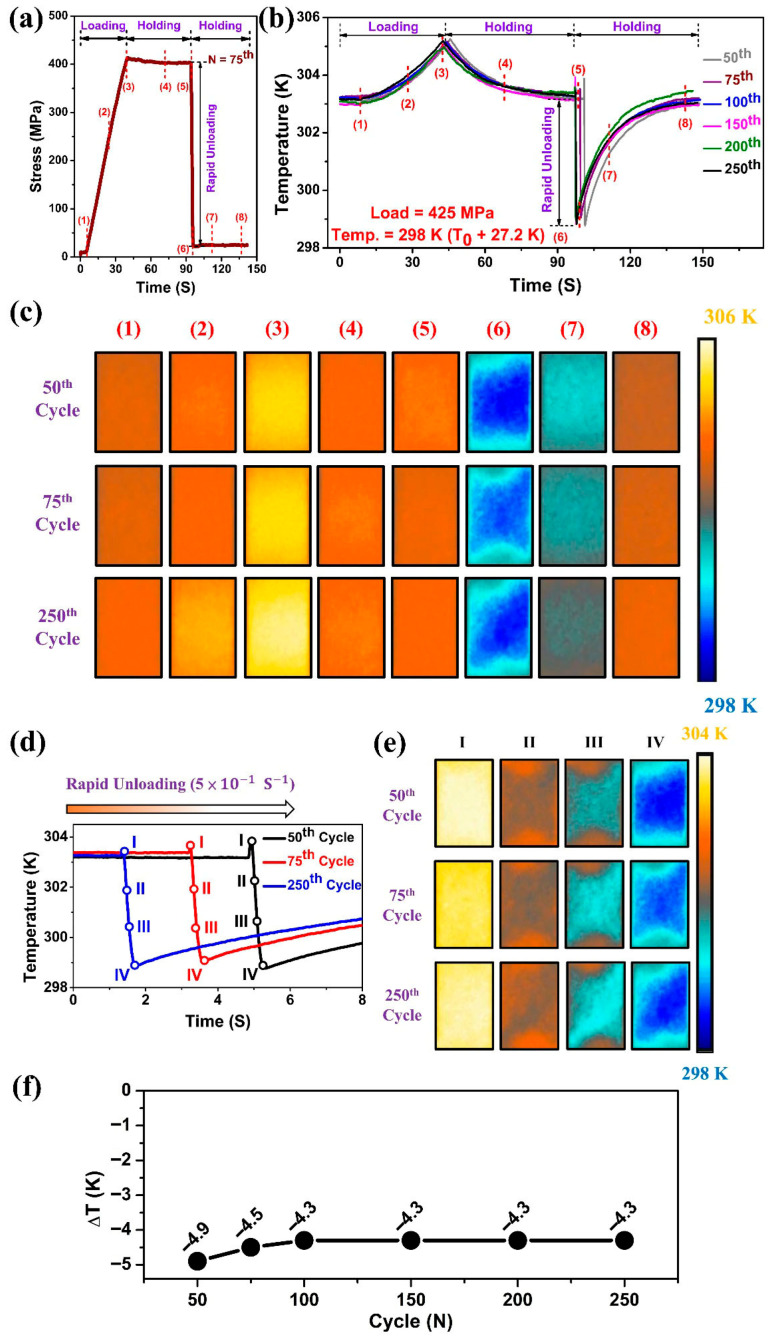
Local temperature profiles of solution treated Ti_50_Ni_41_Cu_7_Co_2_ SMA at 425 MPa during tensile stretching cycles; (**a**) stress vs. time plot, (**b**) temperature vs. time plot and (**c**) corresponding sequential temperature profiles during load and unloading at tensile stretching cycles of 50th, 75th and 250th; local temperature profiles at tensile stretching cycles of 50th, 75th and 250th just at rapid unloading (**d**) temperature vs. time plot, and (**e**) corresponding temperature profiles; (**f**) evolution of elastocaloric cooling effect with cycling number.

According to the superelasticity results shown in Section 3.3, the superelastic behavior became stable after several cycles. Therefore, the initial cycles were ignored, and only the elastocaloric cooling effect from the 50th to the 250th cycle was considered. Figure 5 shows the local temperature profiles captured by the IR camera at different tensile cycles with a loading stress of 425 MPa. The steps involved in measuring the elastocaloric cooling effect of Ti_50_Ni_41_Cu_7_Co_2_ SMA are presented in Figure 5a. During loading, a slow strain rate of 3 × 10^−4^ s^−1^ was applied until a maximum load of 425 MPa was reached. Thereafter, the load was held for 50 s, and then rapidly unloaded to 50 MPa with a high strain rate of 5 × 10^−1^ s^−1^ to achieve a near adiabatic condition during the reverse phase transformation [26].

The average temperature change with respect to time during loading and unloading (both forward and reverse transformation) over an observation area of 8 × 8 mm^2^ is shown in Figure 5b. To study the localized energy absorption and release during loading and unloading, sequential points were identified and marked on the curves, and their corresponding thermographs at specific cycles were presented in Figure 5c. Point 1 was identified as the beginning of the martensitic transformation (B2 → B19′), as a slight rise in temperature was observed after this point. As the load increased, the temperature of the sample rose, and the temperature field (points 2 & 3) became slightly nonuniform. As can be seen in Figure 3c,c’, a hot zone appeared near the center of the specimen, where higher deformation was observed, corresponding to the preferred region for phase transformation. Additionally, the upper and lower regions of the sample, which touch the clamps, also contribute to heat transfer, resulting in lower temperatures in these portions. Point 3 is marked at the peak of the temperature–time curve, where the load reached a maximum stress of 425 MPa. After reaching a maximum load of 425 MPa, the sample was held in the strained condition for 50 s, allowing the sample temperature to reach ambient temperature through heat exchange (Points 4 & 5).

The reverse transformation started immediately upon unloading from Point 5. The temperature of the sample dropped rapidly by −4.9 K for the 50th cycle and −4.3 K for the 250th cycle, respectively, due to the elastocaloric effect induced by the reverse transformation. Finally, heat transfer between the specimen and the surrounding environment raised the temperature of the specimen, exhibiting a uniform temperature profile (Figure 5c—Points 7 and 8). Figure 5d,e show the detailed evolution of temperature distributions during the unloading process at the 50th, 75th, and 250th cycles. The generation and propagation of the cold bands caused by the reverse martensitic transformation during rapid unloading were observed. The temperature of the samples dropped rapidly with the expansion of the cold bands, resulting in stable elastocaloric profiles over 250 cycles.

The evolution of ΔT with cycle number of the Ti_50_Ni_41_Cu_7_Co_2_ SMA is shown in Figure 5f. It has been observed that for the 50th cycle, the ΔT was higher (−4.9 K); however, with an increase in testing cycles, the ΔT decreased slightly and stabilized at −4.3 K by the 100th cycle. Beyond the 100th cycle, the elastocaloric effect became stable, indicating no further changes in microstructure during loading.

With an increased number of repeated cycles, the cooling ability decreased slightly from −4.9 K at the 50th cycle to an almost stable cooling capacity of −4.3 K, while the temperature profile repeatedly followed the same pattern during loading and unloading of cycles. Additionally, the trained (cycled) Ti_50_Ni_41_Cu_7_Co_2_ SMA exhibited uniform temperature profiles, which could serve efficiently for elastocaloric applications.

### 3.5. Study of Local Transformation Behavior After 250 Superelastic Cycles

**Figure 6:**
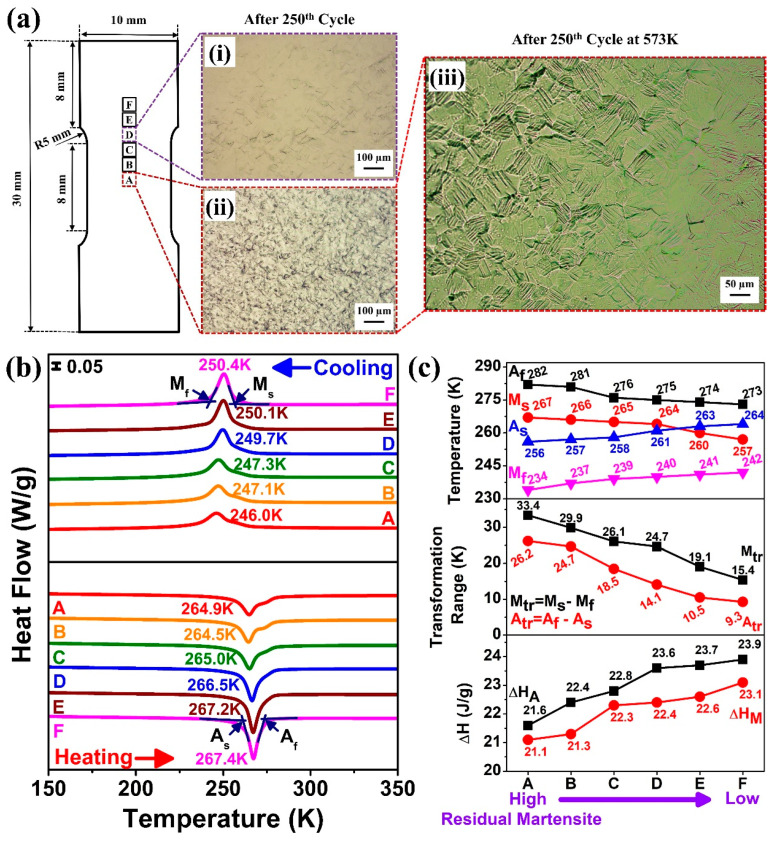
Results at different regions of solution treated Ti_50_Ni_41_Cu_7_Co_2_ SMA tensile specimen after the 250th tensile stretching cycle; (**a**) optical microscope image of (**i**) region D, (**ii**) region A, (**iii**) region A reheated at 573 K after the 250th tensile stretching cycle; DSC results (**b**) cooling & heating heat flow curves and (**c**) phase transition temperature, phase transition temperature ranges and latent heat change curves.

A systematic study was conducted to reveal the local transformation behavior over the tensile specimen after the 250th superelastic cycle. The tensile specimen was sectioned in different regions (A–F) as shown in Figure 6a. Figure 6(ai) shows the optical microscope (OM) image of region D (far away from the deformation zone), and Figure 6(aii) shows the OM image of region A (at the deformation zone). A small amount of martensite appeared in region D, characterized by less strain variation within this region; however, region A underwent greater deformation, resulting in more residual martensite. Figure 6(aiii) shows the OM image of region A after heating at 573 K. The retained martensite indicates that defects generated during cyclic superelastic loading stabilized the martensitic phase, preventing its reversion to austenite even well above A_f_ (282 K), as shown in Figure 6a. This stabilization resulted in reduced strain after the cyclic test, as shown in Figure 3 and Figure 4.

Figure 6b shows the DSC results for regions A–F of the tensile specimen. During cooling, the phase transformation signal (B2 → B19′) flattened gradually as the position moved from region F to A (increasing residual martensite), with decreases in the peak transformation temperature (from 250.4 K to 246.0 K). Similar behavior was observed while heating; the reverse martensitic phase peak (B19′ → B2) flattened similarly, and the reverse peak transformation temperature also decreased from 267.4 K to 264.9 K. It is obvious from the DIC experimental result that closer to region A, higher residual martensite and dislocation formed, which suppressed the phase transformation, resembling the effect of cold working [27].

Figure 6c summarizes the phase transition temperatures across regions A–F. As moved from region F to A (increasing residual martensite), M_s_ and A_f_ temperatures increased from 257 K to 267 K and 273 K to 282 K, respectively. On the other hand, A_s_ and M_f_ temperatures decreased from 264 K to 256 K and from 252 K to 234 K, respectively. Due to the heavy processing near the deformation zone, M_s_ and A_f_ rose while M_f_ and A_s_ fell, and hence the transformation peak broadened. Further, the transition temperature ranges, i.e., M_tr_ and A_tr_, increased from 15.4 K to 33.4 K and from 9.3 K to 26.2 K, respectively, across regions from F to A (i.e., increasing residual martensite). The wider ranges near region A result from defects generated during superelastic cycles, which created stress fields that alter local martensitic transformation temperatures [28]. It was noted that the increase in M_s_ but decrease in M_f_ suggested that some martensitic transformation was triggered, but some was hindered by the dislocations. Figure 6c also includes the latent heat variations across the tensile specimen. From region F to A (increasing residual martensite), both ΔH_M_ and ΔH_A_ decreased from 23.1 to 21.1 J/g and 23.9 to 21.6 J/g, respectively. This indicated that dislocations generated during the cyclic test suppressed martensitic transformation, resulting in smaller latent heat. Compared to region F, the highly deformed region A exhibited higher A_f_ and M_s_, lower A_s_ & M_f_, and wider M_tr_ and A_tr_ transformation temperature range with reduced ΔH_M_ and ΔH_A_. These observations confirmed the stabilization and hindrance of martensitic phase transformation due to the generation of dislocations during superelastic cycles.

### 3.6. Effect of Operating Temperature on Superelasticity and Elastocaloric Effects

**Figure 7:**
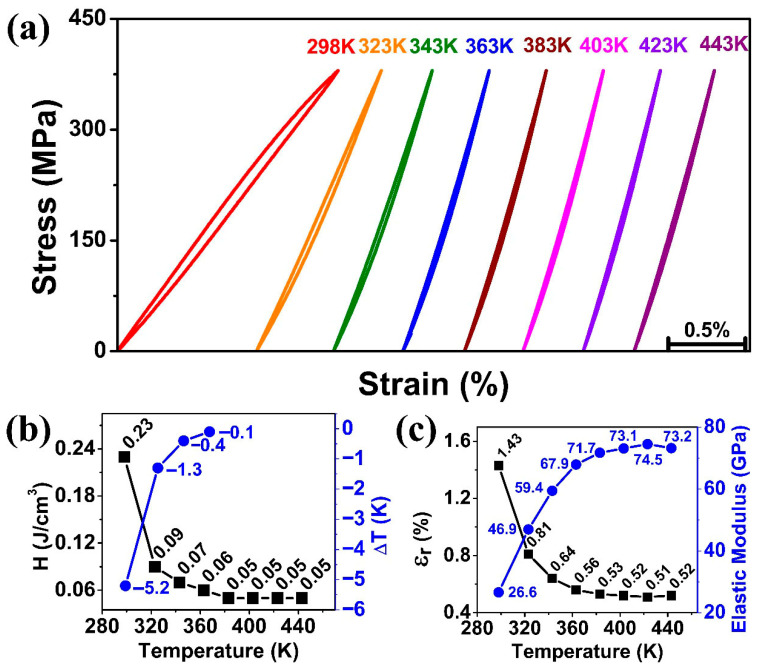
Effect of operating temperatures on the 100 cycle-trained Ti_50_Ni_41_Cu_7_Co_2_ SMA (**a**) Superelastic curves, (**b**) Dissipated energy and elastocaloric effect, and (**c**) Recoverable strain and elastic modulus.

In order to serve industrial requirements, a shape memory alloy with stable superelasticity and elastocaloric cooling effect over an operating temperature range is desired. According to the results shown in Section 3.3 and Section 3.4, the SMAs showed high stability in superelasticity and elastocaloric cooling effect after 50 superelastic cycles. Therefore, a tensile sample trained with 100 superelastic cycles was selected for studying the effect of operating temperature on its functional performance. Figure 7 shows the effect of operating temperature on superelasticity and elastocaloric effect tested between 298 K and 443 K. Under the maximum stress of 425 MPa, the stress–strain curves remained linear for all temperatures, as shown in Figure 7a.

Figure 7b shows the energy dissipation of the stress–strain curves at different temperatures, which dropped drastically for the early increase in temperature. It was 0.23 J/cm^3^ at 298 K and dropped to 0.09 J/cm^3^ at 323 K, and became almost stable at 0.05 J/cm^3^ beyond 363 K. Figure 7b also shows the elastocaloric cooling effect at different temperatures. While the highest cooling effect was measured at 298 K (−5.2 K), and then sudden drop in cooling effect (dropped by 75%) was measured at 323 K, which was −1.3 K. Thereafter, elastocaloric effects continuously decreased to −0.4 K and −0.1 K at temperatures of 343 K and 363 K, respectively. After 383 K, no obvious elastocaloric effect was observed, suggesting that mainly elastic deformation occurred at the temperature of 383 K. These features followed the Clausius–Clapeyron relation, which meant that the more the operating temperature is away from the austenite finish temperature (A_f_), the less likely it is to produce stress-induced martensitic transformation.

Figure 7c shows the relationship between the recoverable strain (ε_r_) and elastic modulus (E) at different operating temperatures. For all the temperature conditions, ε_irr_ was negligible, and ε_t_ and ε_r_ were equal. The recoverable strain (ε_r_) almost followed a similar trend as that of the energy dissipation, showing a rapid decline from 1.43% at 298 K, to 0.81% at 323 K, and to just 0.52% above 363 K. Additionally, the elastic modulus was calculated using Equation (1), according to S. Zhao et al. [29] as follows:

(1) $$E=\frac{\sigma_{max}}{\varepsilon_{max}}$$

where *E*, *σ_max_*, and *ε_max_* represent the elastic modulus, the maximum stress, and the maximum strain, respectively. The elastic modulus rose drastically from 26.6 GPa to 71.7 GPa with increasing temperature from 298 K to 383 K, and then became stable. Above the operating temperature of 383 K, no obvious phase transformation was detected (neither energy dissipation nor elastocaloric effect), which suggested the stress of 425 MPa was not sufficient for stress-induced martensitic transformation at these high temperatures (according to the Clausius–Clapeyron relation), and hence only elastic deformation occurred in the specimen. Therefore, the elastic moduli at temperatures of 383 K, 403 K, 423 K, and 443 K were 71.7 GPa, 73.1 GPa, 74.5 GPa, and 73.2 GPa, respectively, which are quite close to each other and provide almost stable elastic modulus for high-temperature conditions. It is also noted that, even though the martensitic transformation was limited at these high temperatures, relatively large recoverable strains (>0.5%) were available, surpassing the 0.2% plastic deformation strain for conventional metallic materials.

After being trained with 100 superelastic cycles, the Ti_50_Ni_41_Cu_7_Co_2_ SMA showed a rapid decline in the performance of the elastocaloric effect with an increase in operating temperature due to the insufficient stress to induce martensitic transformation. It is expected that, to trigger the superelasticity and elastocaloric effect at high operating temperatures, an increase in the applied stress is needed. However, an increase in applied stress is also expected to disrupt the original stability and may cause the formation of residual strain, which needs further study in the future.

### 3.7. Discussion on the Performance of TiNiCuCo SMA

The Ti_50_Ni_41_Cu_7_Co_2_ SMA exhibits stable functional properties after superelastic training, including a 1.3% recoverable strain, a −4.3 K elastocaloric cooling effect, and superelasticity over a wide temperature range with strains exceeding 0.5%. The stabilization of functionality is also observed in a binary TiNi_50.8_ SMA [29], which exhibits reversible strains of around 1.6–2.3% after 100 cycles. This quasi-linear feature was also observed in compression deformation of TiNi_50.4_ [30] and Ti_50_Ni_48_Fe_2_ [31] SMAs, in which recoverable strains of about 2% and 1.6% were reported, respectively. The smaller recoverable strain in the TiNiCuCo shape memory alloy was attributed to the addition of copper, which is known to reduce recoverable strain as the copper content increased [31]. Additionally, the TiNi_50.4_ [30] and Ti_50_Ni_48_Fe_2_ [31] SMAs exhibited elastocaloric cooling effects of −3.9 K and −3.5 K after cyclic loading, respectively, which were slightly smaller than that of the Ti_50_Ni_41_Cu_7_Co_2_ SMA reported in this study. These features indicated that the Ti_50_Ni_41_Cu_7_Co_2_ SMA could generate a comparable or better elastocaloric cooling effect with a smaller strain, which is beneficial for designing a compact cooling system.

The as-homogenized Ti-rich Ti_54_Ni_31.7_Cu_12.3_Co_2_ bulk SMA was also reported to exhibit quasi-linear superelastic behavior after 100 cycles [14]. Due to the difference in the testing method, where the sample was unloaded to 100 MPa (not full unloading) and then reloaded, their results could not be directly compared with the functional properties reported here. Nevertheless, a trend was that the Ti_50_Ni_41_Cu_7_Co_2_ SMA exhibited much better stability during the cyclic loading at the solution-treated state during the superelastic cycles. Furthermore, since the shape memory effect, the elastocaloric effect, and the operating temperature window were not tested for the Ti_54_Ni_31.7_Cu_12.3_Co_2_ SMA [14], this study provides a detailed analysis of the bulk TiNiCuCo SMA.

Excellent functional stability over 107 cycles was reported in the TiNiCuCo SMAs in the thin-film form [13,32]. Owing to the refined grain size and nano-scale Ti_2_Cu phase formed in the thin films, the strength and stability of the TiNiCuCo SMAs were significantly improved. However, as shown in the previous study [14] and this one, the bulk Ti_54_Ni_31.7_Cu_12.3_Co_2_ and Ti_50_Ni_41_Cu_7_Co_2_ SMAs did not demonstrate results as promising as their thin-film counterparts. Therefore, for applications of TiNiCuCo SMAs in bulk form, careful control of the microstructure is necessary to further enhance their functional stability, such as through cold working, grain refinement, or precipitation hardening (achieved through alloy design). Since studies on bulk TiNiCuCo SMAs are limited, this study provides a comprehensive analysis of the functional performance of the Ti_50_Ni_41_Cu_7_Co_2_ SMA in its solution-treated state. Further improvements in performance are expected to be realized through alloy design and microstructure control.

## 4. Conclusions

This study systematically investigates the functional properties of a bulk Ti_50_Ni_41_Cu_7_Co_2_ SMA in the solution-treated state. The alloy exhibited a M_s_ temperature of 250 K with a small hysteresis of 14 K. It demonstrated excellent SME, reaching 6.21% recoverable strain with 0.44% irrecoverable strain at a bias stress of 300 MPa. Cyclic tensile loading transformed the flag-shaped superelastic curve into a quasi-linear one, and the initial nonuniform local strain bands became more homogeneous with cycling. After the cyclic superelastic tests, the transformation strain, transformation stress, and elastocaloric cooling effect became stable at 1.3%, 270 MPa, and −4.3 K, respectively. For the trained sample, the elastocaloric effect decreased quickly with rising operating temperature and disappeared above 383 K. The recoverable strain (ε_r_) showed a similar decline, stabilizing at 0.52% beyond 363 K. In contrast, the elastic modulus increased sharply from 26.6 GPa to 73.2 GPa, and became stable above 403 K. Notably, a recoverable strain of ~0.5% was still achievable up to 443 K.

## Figures and Tables

**Table 1:** EDS composition analyses of the ST Ti_50_Ni_41_Cu_7_Co_2_ sample.

Location	Composition (at. %)			
	Ti	Ni	Cu	Co
A	65.9	28.8	4.5	0.8
B	64.9	29.4	4.7	1.0
C	65.5	29.0	4.6	0.9
D	49.3	40.2	8.6	1.9
E	48.9	40.6	8.8	1.7
F	48.8	40.6	8.9	1.7

## Data Availability

The original contributions presented in this study are included in the article. Further inquiries can be directed to the corresponding authors.

## Abbreviations

The following abbreviations are used in this manuscript:

SMA	Shape memory alloy
SE	Superelasticity
ECE	Elastocaloric cooling effect
SME	Shape memory effect
SEM	Scanning Electron Microscopy
DSC	Differential Scanning Calorimeter
DMA	Dynamic mechanical analyzer
DIC	Digital image correlation
M_s_	Martensite start temperature
M_p_	Martensite peak temperature
M_f_	Martensite finish temperature
A_s_	Austenite start temperature
A_p_	Austenite peak temperature
A_f_	Austenite finish temperature
